# Functional and genomic characterization of three novel cell lines derived from a metastatic gallbladder cancer tumor

**DOI:** 10.1186/s40659-020-00282-7

**Published:** 2020-04-15

**Authors:** Patricia García, Carolina Bizama, Lorena Rosa, Jaime A. Espinoza, Helga Weber, Javier Cerda-Infante, Marianela Sánchez, Viviana P. Montecinos, Justo Lorenzo-Bermejo, Felix Boekstegers, Marcela Dávila-López, Francisca Alfaro, Claudia Leiva-Acevedo, Zasha Parra, Diego Romero, Sumie Kato, Pamela Leal, Marcela Lagos, Juan Carlos Roa

**Affiliations:** 1 Department of Pathology, Faculty of Medicine, Pontificia Universidad Católica de Chile, Santiago, Chile; 2 Applied Molecular and Cellular Biology PhD Program, Universidad de La Frontera, Temuco, Chile; 3 Science for Life Laboratory, Division of Genome Biology, Department of Medical Biochemistry and Biophysics, Karolinska Institutet, Stockholm, Sweden; 4 Center of Excellence in Translational Medicine (CEMT) and Scientific and Technological Bioresource Nucleus (BIOREN), Universidad de La Frontera, Temuco, Chile; 5 Department of Hematology Oncology; Cellular and Molecular Biology, Pontificia Universidad Católica de Chile, Santiago, Chile; 6 Department of Hematology Oncology, Pontificia Universidad Católica de Chile, Santiago, Chile; 7 Statistical Genetics Research Group, Institute of Medical Biometry and Informatics, University of Heidelberg, Heidelberg, Germany; 8 Bioinformatics Core Facility, Sahlgrenska Academy, University of Gothenburg, Gothenburg, Sweden; 9 Cytogenetics Laboratory, Complejo Asistencial Dr. Sótero del Río, Santiago, Chile; 10 Division of Obstetrics and Gynecology, Faculty of Medicine, Pontificia Universidad Católica de Chile, Santiago, Chile; 11 Department of Clinical Laboratory, Faculty of Medicine, Pontificia Universidad Católica de Chile, Santiago, Chile; 12 Department of Pathology, Faculty of Medicine, Millennium Institute of Immunology and Immunotherapy, Pontificia Universidad Católica de Chile, Santiago, Chile

**Keywords:** Gallbladder cancer, Cancer cell lines, Ascites, Native American ancestry, Gene expression profile

## Abstract

**Background:**

Gallbladder cancer (GBC) is the most common tumor of the biliary tract. The incidence of GBC shows a large geographic variability, being particularly frequent in Native American populations. In Chile, GBC represents the second cause of cancer-related death among women. We describe here the establishment of three novel cell lines derived from the ascitic fluid of a Chilean GBC patient, who presented 46% European, 36% Mapuche, 12% Aymara and 6% African ancestry.

**Results:**

After immunocytochemical staining of the primary cell culture, we isolated and comprehensively characterized three independent clones (PUC-GBC1, PUC-GBC2 and PUC-GBC3) by short tandem repeat DNA profiling and RNA sequencing as well as karyotype, doubling time, chemosensitivity, in vitro migration capability and in vivo tumorigenicity assay. Primary culture cells showed high expression of CK7, CK19, CA 19-9, MUC1 and MUC16, and negative expression of mesothelial markers. The three isolated clones displayed an epithelial phenotype and an abnormal structure and number of chromosomes. RNA sequencing confirmed the increased expression of cytokeratin and mucin genes, and also of *TP53* and *ERBB2* with some differences among the three cells lines, and revealed a novel exonic mutation in *NF1*. The PUC-GBC3 clone was the most aggressive according to histopathological features and the tumorigenic capacity in NSG mice.

**Conclusions:**

The first cell lines established from a Chilean GBC patient represent a new model for studying GBC in patients of Native American descent.

## Background

Gallbladder cancer (GBC; ICD-10 diagnosis code C23) accounts for 80–95% of biliary tract cancers and is the sixth most common gastrointestinal cancer worldwide [1, 2]. This aggressive disease is relative rare in most high-income countries, but GBC affects at least 219.420 persons every year worldwide. The geographic areas with the highest mortality rates include Chile, Bolivia, Korea, Nepal, Bangladesh, Japan, Peru, Czech Republic and Slovakia [3]. In Chile, GBC is one of the most common neoplasms and represents the second cause of cancer-related death in women, the mortality rate in 2015 was 10.2 deaths per 100 000 women [4]. The regional distribution of GBC in Chile is correlated with the prevalence of its main risk factor, gallstone disease, together with the regional proportions of Mapuche Native American ancestry and other socioeconomic factors [5, 6].

One of the major characteristics of GBC is its late diagnosis and ineffective treatment [7]. The lack of specific symptoms in early stages and effective diagnostic biomarkers results in most patients being diagnosed with locally advanced or metastatic disease [8, 9]. Unfortunately, the 5-year survival rate is less than 10% for these patients [10].

To improve the clinical prognosis of GBC patients, more efficient methods for early diagnosis and more effective therapeutic approaches must be developed. Research on GBC is challenging not only because it is a rare tumor worldwide, but also due to the difficulty of accessing human tissue samples and the lack of experimental models. Obtaining fresh frozen tissue samples is extremely demanding because most of the early lesions are thin, flat, incidental and masked with gross acute inflammatory changes, and chemotherapy and palliative care is frequently offered to patients with advanced tumor after diagnosis confirmation by fine needle aspiration biopsy [11]. The prophylactic removal of gallbladders with stones hampers the study of the natural history of GBC, and the non-existence of animal models mimicking the progression of pre-neoplastic lesions to invasive cancer translates into the present basic and clinical know-how relying on other types of gastrointestinal cancer.

A large proportion of the current knowledge on GBC biology comes from studies performed using GBC cell lines, which have played a critical role in identifying therapeutic targets and facilitating the rapid screening of new anticancer compounds. Most GBC cell lines derive from primary tumors and metastatic sites and have been isolated from Japanese [12–16], Korean [17] and Chinese [18, 19] patients. Cell lines from GBC patients with Native American ancestry are not available yet.

To our knowledge, this is the first report on the development and characterization of cell lines derived from a GBC patient of Native American descent. We describe the isolation of three cell lines from the ascites of a single patient, which conserved the genomic features and malignant behavior of the primary GBC tumor. Our finding suggest that the three established cell lines reflect the heterogeneity of GBC tumors and provide a useful model to investigate the mechanisms underlying late stage GBC progression and metastasis, gain new insight into drug resistance mechanisms and test new therapeutic strategies.

## Results

### Establishment of GBC cell lines from one primary cell culture

**Fig. 1:**
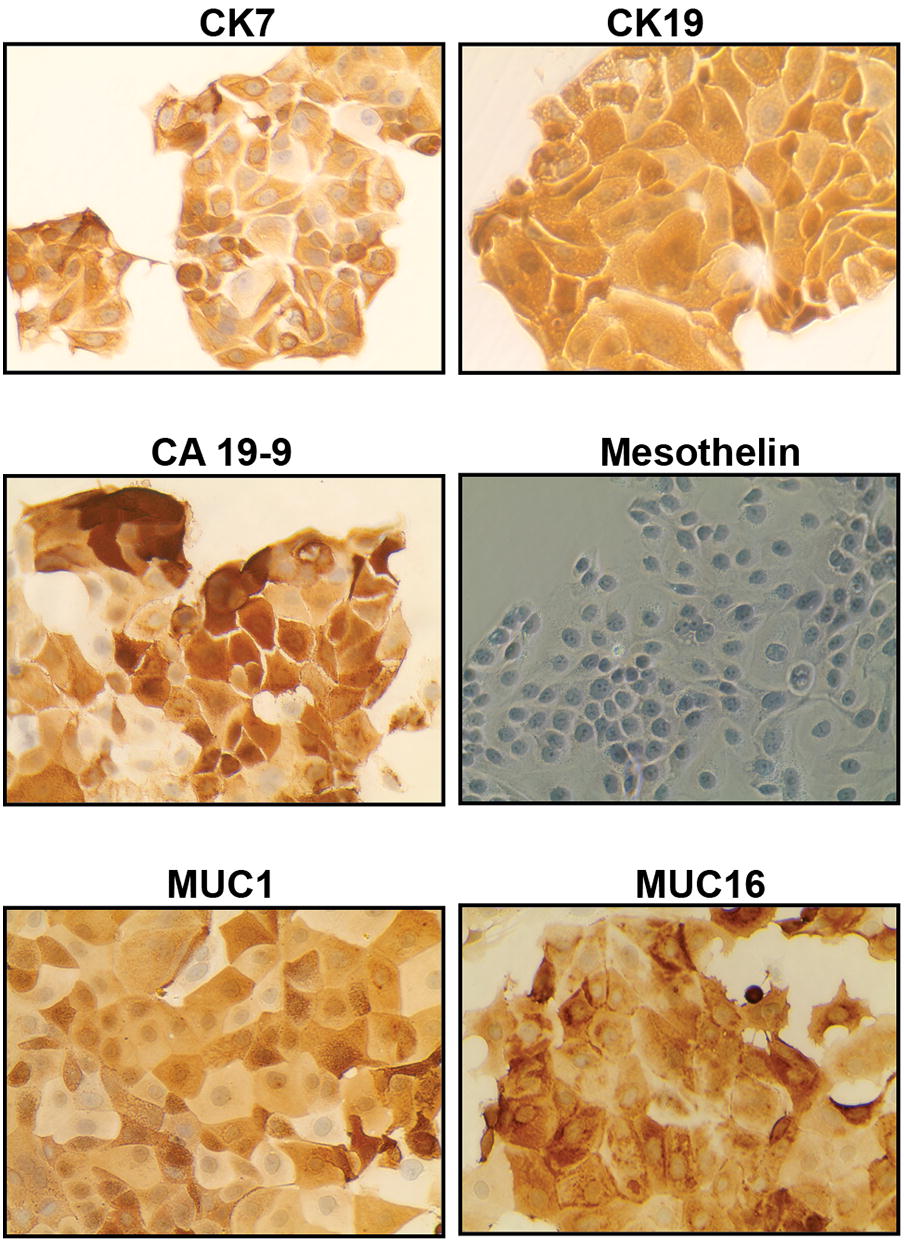
Ascites-derived primary culture has an epithelial phenotype. Representative micrographs showing the expression of characteristic epithelial, mesenchymal and tumor markers in the ascites-derived primary culture. Cells showed strong positive staining for CK7, CK19, CA 19-9, MUC1 and MUC16, whereas mesothelin was absent. All pictures were taken at ×40 magnification

We successfully established a primary culture of tumor cells derived from the ascitic fluid of a 60-year-old man diagnosed with metastatic GBC, who showed the following percentages of genetic ancestry: 46% European, 36% Mapuche Native American, 12% Aymara Native American and 6% African. Moderate to strong expression (2+ to 3+) of the epithelial markers CK7 (cytokeratin 7) and CK19 (cytokeratin 19) was observed in 100% of the ascites-derived cells, mesothelin was not expressed. Almost 90% of the cells showed high expression levels (3+) of the tumor carbohydrate antigen 19-9 (CA 19-9) and moderate expression (2+) of the mucins MUC1 (mucin 1) and MUC16 (mucin 16) (Fig. 1). Taken together, all these markers indicate an epithelial origin of the primary culture.

**Fig. 2:**
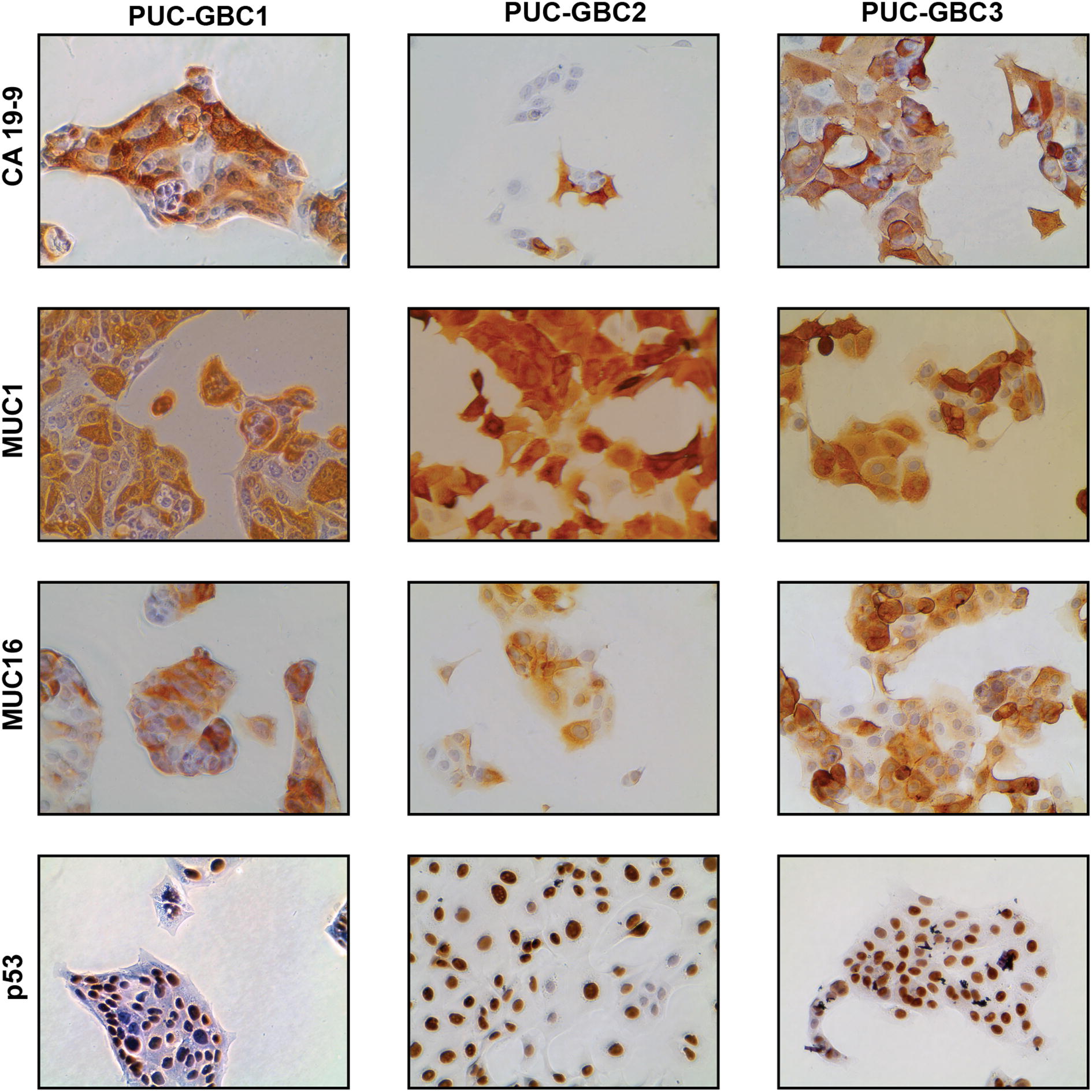
All three cell lines retain the epithelial phenotype of the primary culture. Representative micrographs of immunocytochemical staining for CA 19-9, MUC1, MUC16 and p53 in the three clones isolated from the ascites-derived primary culture. All pictures were taken at ×40 magnification

The primary culture consisted of epithelial cells with different morphologies, which grew as heterogeneous cell populations. In order to generate monoclonal cell lines, we used a limiting dilution procedure to isolate and spread individual cells. We established three cell lines as separate cultures, namely PUC-GBC1, PUC-GBC2 and PUC-GBC3. In consistency with the primary culture, the newly established cell lines showed high expression levels of the epithelial markers CK7 and CK19 (3+, 2+) and no mesothelin expression (see Additional file 1). As depicted in Fig. 2, the three cell lines showed positive staining for CA 19-9, MUC1 and MUC16, with moderate to strong (2+ to 3+) intensity in more than 80% of tumor cells, with the exception of CA 19-9, which was only expressed in 20% of PUC-GBC2 cells. We also evaluated the protein expression of the tumor suppressor p53 since its stabilization/activation has been associated with mutations in the *TP53* (Tumor Protein P53) gene and poor prognosis of GBC [20–25]. All three cell lines were p53 positive, with a strong brown nuclear staining in 100% of the tumor cells.

### Short tandem repeat (STR) profiling, chromosome analyses and RNA sequencing

The STR DNA profile analysis revealed very minor discrepancies among the three cell lines: only PUC-GBC3 lost one allele at D7S820 (Table 1).

**Table 1:** Short tandem repeat (STR) DNA profiling of ascites-derived gallbladder cancer cell lines

STR marker	Allelle(s)		
	PUC-GBC1	PUC-GBC2	PUC-GBC3
AMEL	X Y	X Y	X Y
CSF1PO	10 11	10 11	10 11
D13S317	11 12	11 12	11 12
D16S539	10 12	10 12	10 12
D21S11	33 2	33 2	33 2
D5S818	7 11	7 11	7 11
D7S820	7 10	7 10	10
TH01	7	7	7
TPOX	8	8	8
vWA	16	16	16

**Fig. 3:**
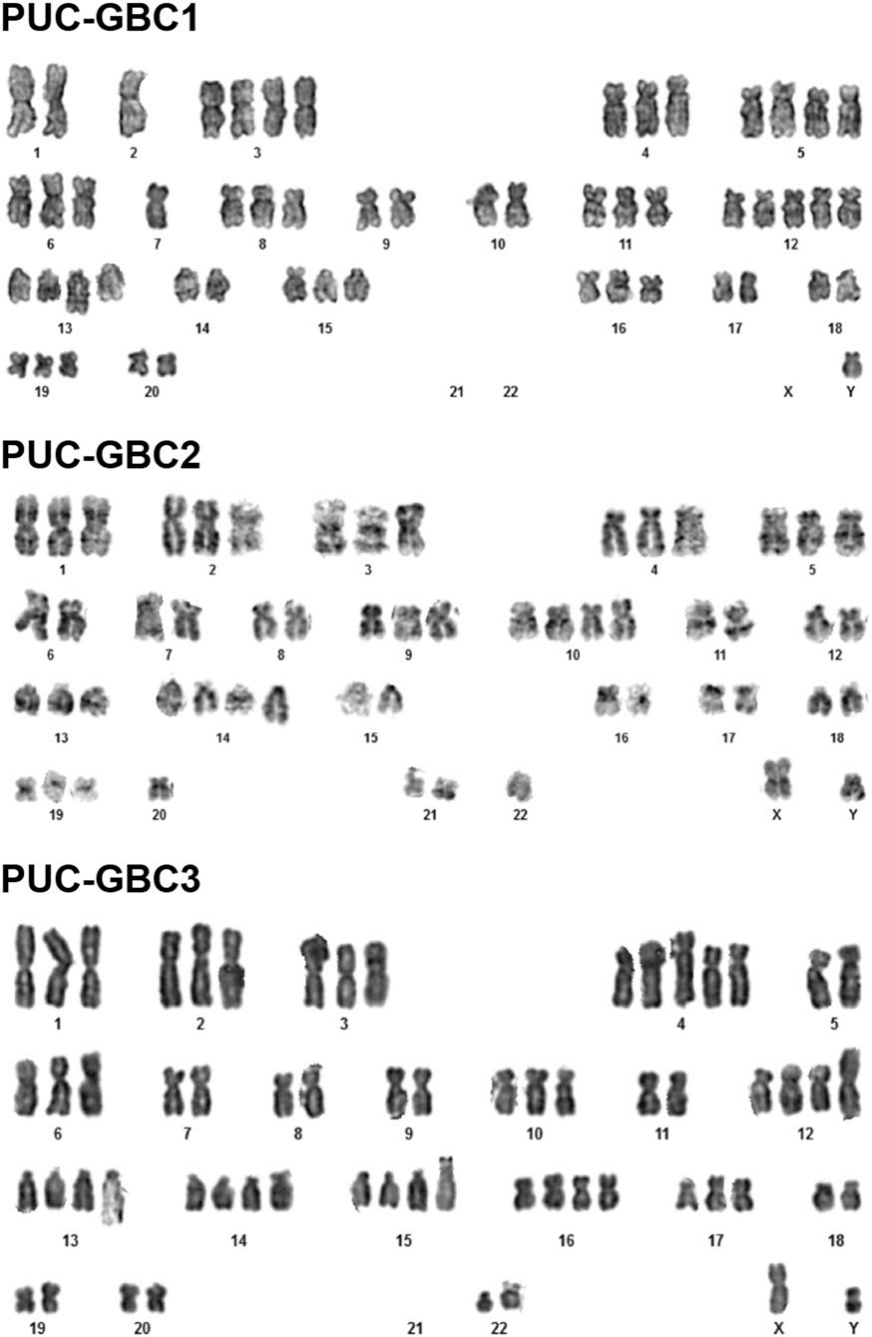
Karyotype analysis of cancer cell lines reveals chromosomal heterogeneity

The modal chromosome number was in the hyperdiploid range (> 50) for the three cell lines. The karyotype for PUC-GBC1 was: XY,-X, +3, +4, +5, +5, −7, +10, +11, +12, +12, +12, +13, add(13)(q33), +14, +15, +16, +16, −17,+ 19, −21, −21, −22, −22. For PUC-GBC2: XY, +1, +2, +3, +4, +5, +9, +10, +10, +13, +14, +14, add(q31), +19, −20, −22 [10]. And for PUC-GBC3: XY, +2, +4, + 4, der(5), −6, −8, −9, −11, i(12)(q10), add(13)(q34), +14, +15 ps+, +16, −18, −19, −20, −21, −21, −21, −22 (Fig. 3).

**Fig. 4:**
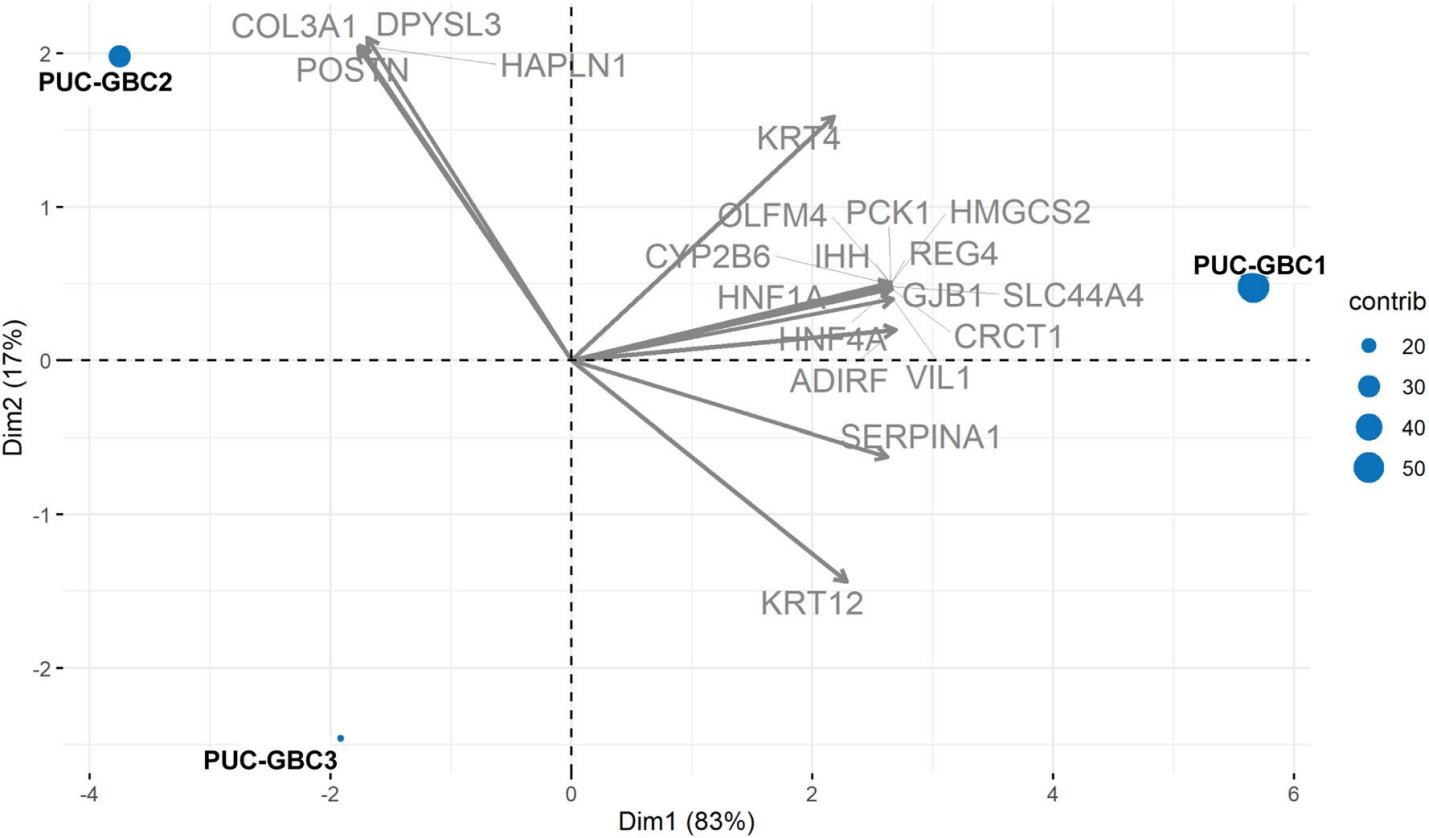
Principal component analysis (PCA) and biplot of the three novel cell lines and the 20 genes with the largest variability in gene expression. Points represent the cell lines, the distance among the points indicates the similarity among the gene expression profiles and arrows show the differentially expressed genes. The proportion of variance explained by each principal component is shown in parentheses

**Fig. 5:**
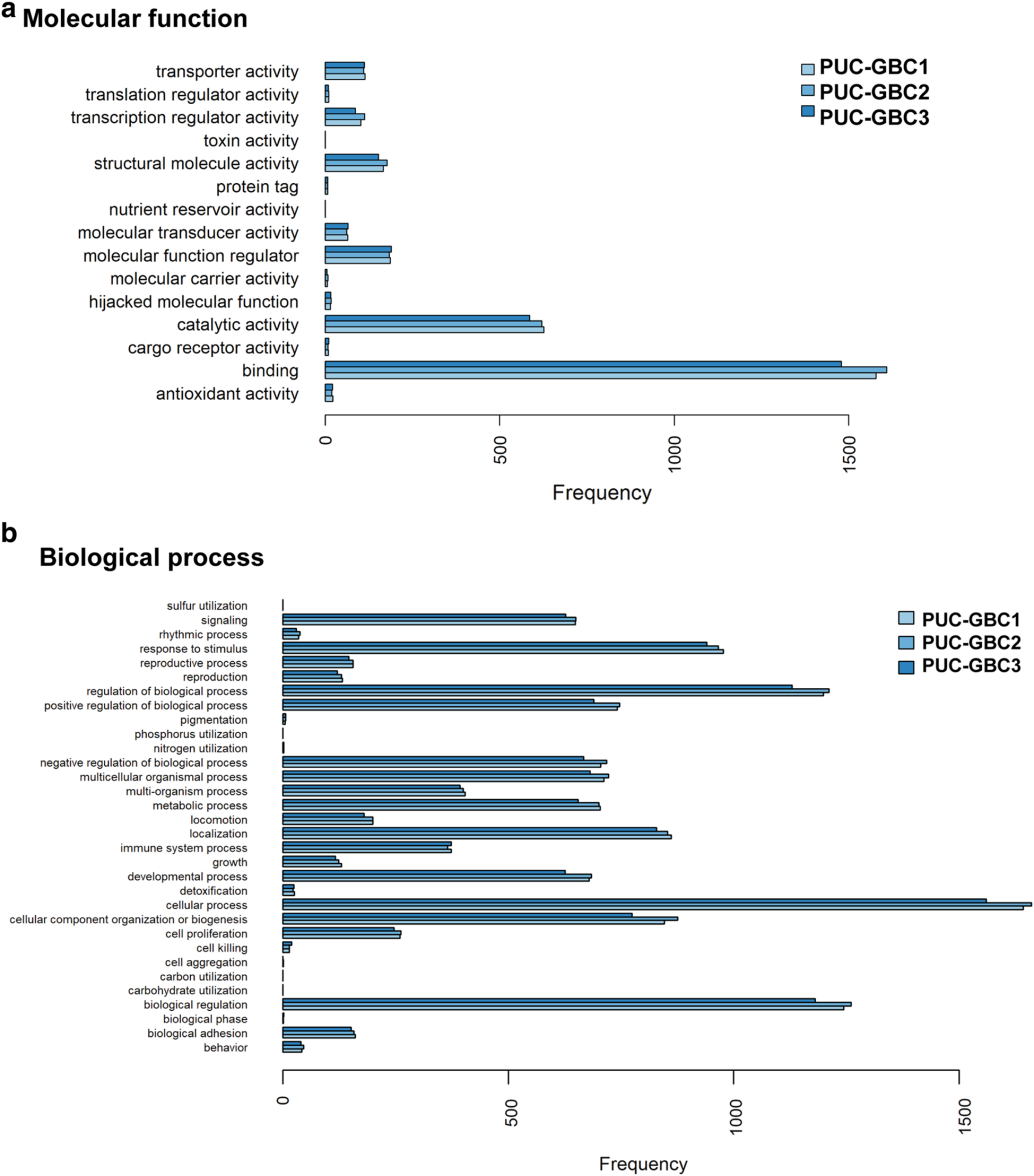
Distribution of level 2 gene ontology (GO) terms including biological process and molecular function among all annotated genes

The analysis of RNA sequencing data confirmed the increased expression of cytokeratin (*KRT7* and *KRT19*) and mucin (*MUC1* and *MUC16*) genes, as well as *TP53* with some differences in expression among the cell lines. For example, *TP53* was weakly expressed in PUC-GBC3. The principal component analysis (PCA) of the three cell lines and the 20 genes with the largest variability in gene expression (highest coefficient of variation) revealed that the expression profiles of PUC-GBC2 and PUC-GBC3 were more similar to each other than to PUC-GBC1 (Fig. 4). Based on the genes with the largest expression variability, PUC-GBC2 showed an enrichment of genes coding for proteins that form the extracellular matrix, such as *COL3A1*, *POSTN* and *HAPLN1*. On the other hand, genes involved in the regulation of cancer stem cell properties (*HNF1A*, *OLFM4*, *PCK1* and *REG4*) and cell metabolism (*CYP2B6* and *SLC44A4*) were overrepresented in PUC-GBC1. Additional file 2 provides the expression values for the three newly established GBC cell lines as normalized transcripts per million. We also performed a gene ontology (GO) enrichment analysis, processing genes in terms of their associated molecular function and biological process. The results showed a similar enrichment of GO terms among the three cell lines (Fig. 5). Binding was the largest sub-category in the molecular function category, followed by catalytic activity, while the major biological processes were cellular process, biological regulation, and regulation of biological process.

According to the MSK-IMPACT database [26], mutations in at least 10% of primary GBC tumors are expected in ten genes: *TP53*, *ATM*, *SMAD4*, *ARID1A*, *CTNNB1*, *NF1*, *NOTCH3*, *PTPRD*, *KEAP1* and *ARID1B*. Among these ten candidate genes, RNA sequencing revealed a novel non-synonymous mutation in *NF1* shared by the three GBC cell lines (chr17:31232174, exon25:c.C3299T). According to SIFT (Sorting Intolerance from Tolerance) and Polyphen-2, this mutation has a damaging amino acid impact (p.S1100L). Additional file 3 lists the number of identified exonic mutations and the annotated genetic variants in the three GBC cell lines. Synonymous variants were more common (n = 5923) than non-synonymous (n = 4368) and frameshift (n = 208), with multiple overlapping variants between the different cell lines (Additional file 4). Considering that these cell lines derived from a metastatic site, genomic alterations were expected in multiple genes, many of them involved in relevant oncogenic signaling pathways. These included non-synonymous single-nucleotide variations (nsSNV) in *CDKN1A* (rs1801270), *MDM4* (rs4252716)*, PIK3CA* (rs2230461), *ERBB2* (rs1058808) and *ERBB3* (rs55699040), a frameshift deletion in *CDKN2A* (NA), among others (see Additional file 3).

### In vitro characterization

**Fig. 6:**
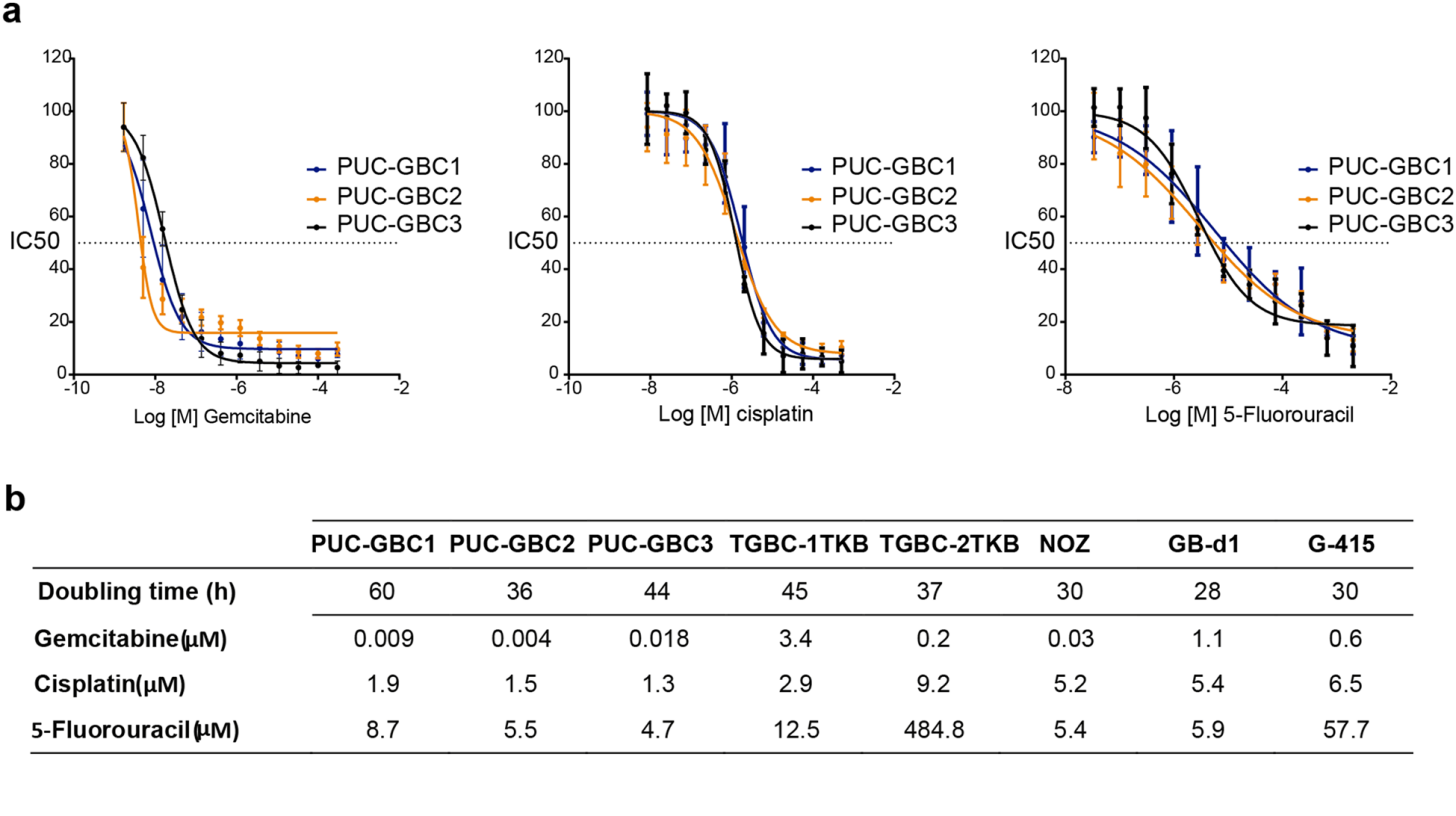
Growth characteristics and chemosensitivity analysis. **a** Dose–response curves of the ascites-derived gallbladder cancer cell lines treated with chemotherapeutic agents. Cells were incubated for 72 h with each drug as single agent before cell viability was assessed via MTS assay. Data are representative of three independent experiments with three technical replicates (mean ± SD) **b** Mean doubling times (hours) and half maximal inhibitory concentration (IC_50_) values of cytotoxic drugs of ascites-derived clones and commercial established GBC cell lines

Growth curves were examined for the three cell lines. The population doubling time was 60 h for PUC-GBC1, 36 h for PUC-GBC2 and 44 h for PUC-GBC3. Representative dose–response curves to gemcitabine, cisplatin and fluorouracil (5-FU), along with IC_50_ values are shown in Fig. 6a. Comparisons with calculated IC_50_ from five commercially available GBC cell lines are shown in Fig. 6b. Our three newly established cell lines showed higher sensitivity to gemcitabine and cisplatin than the commercially available ones. Sensitivity to 5-FU was similar to that shown by GB-d1 and NOZ.

**Fig. 7:**
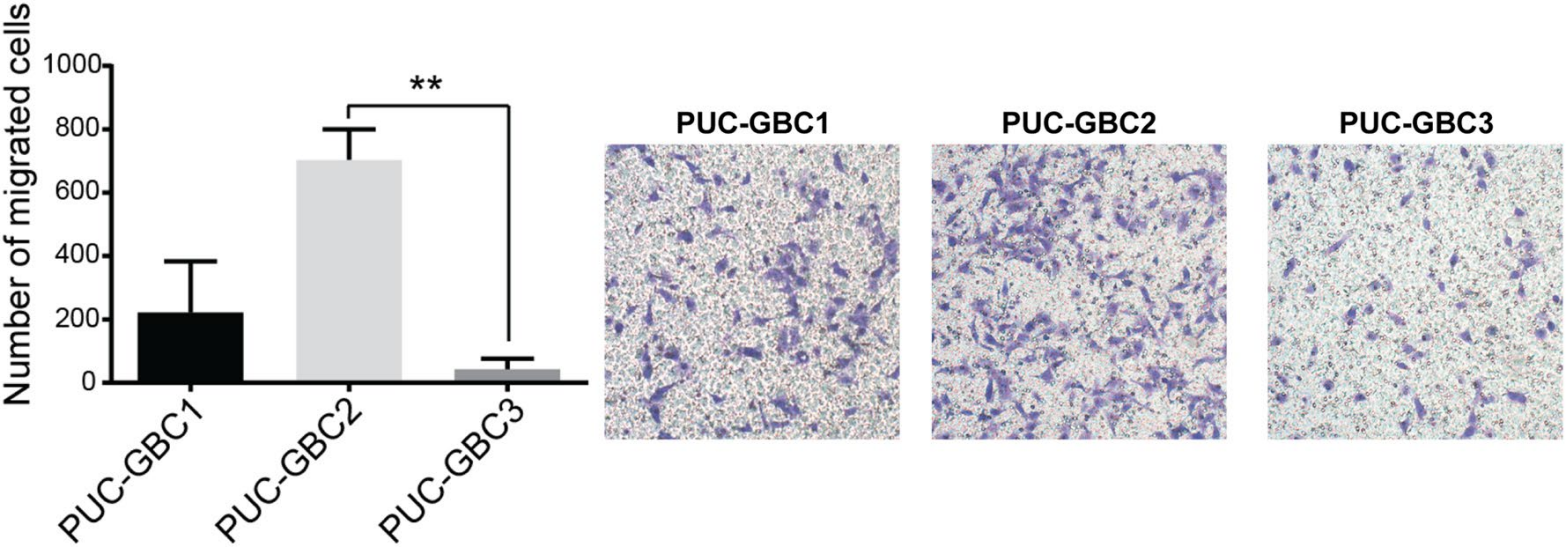
GBC cell lines have differential migratory capacities. Cells were seeded in the upper side of a Transwell^®^ membrane and, after 6 h, migrated cells were stained with crystal violet and counted. PUC-GBC2 had the strongest migration ability compared to PUC-GBC1 and PUC-GBC3. Results are expressed as mean ± SD of 3 independent experiments (**P = 0.0073 by Kruskal–Wallis with Dunn’s post-test)

In vitro migration assays showed that the relative migration rate after 24 h was highest for PUC-GBC2 and lowest for PUC-GBC3 (*P* = 0.0073) (Fig. 7).

### Tumorigenic potential

**Fig. 8:**
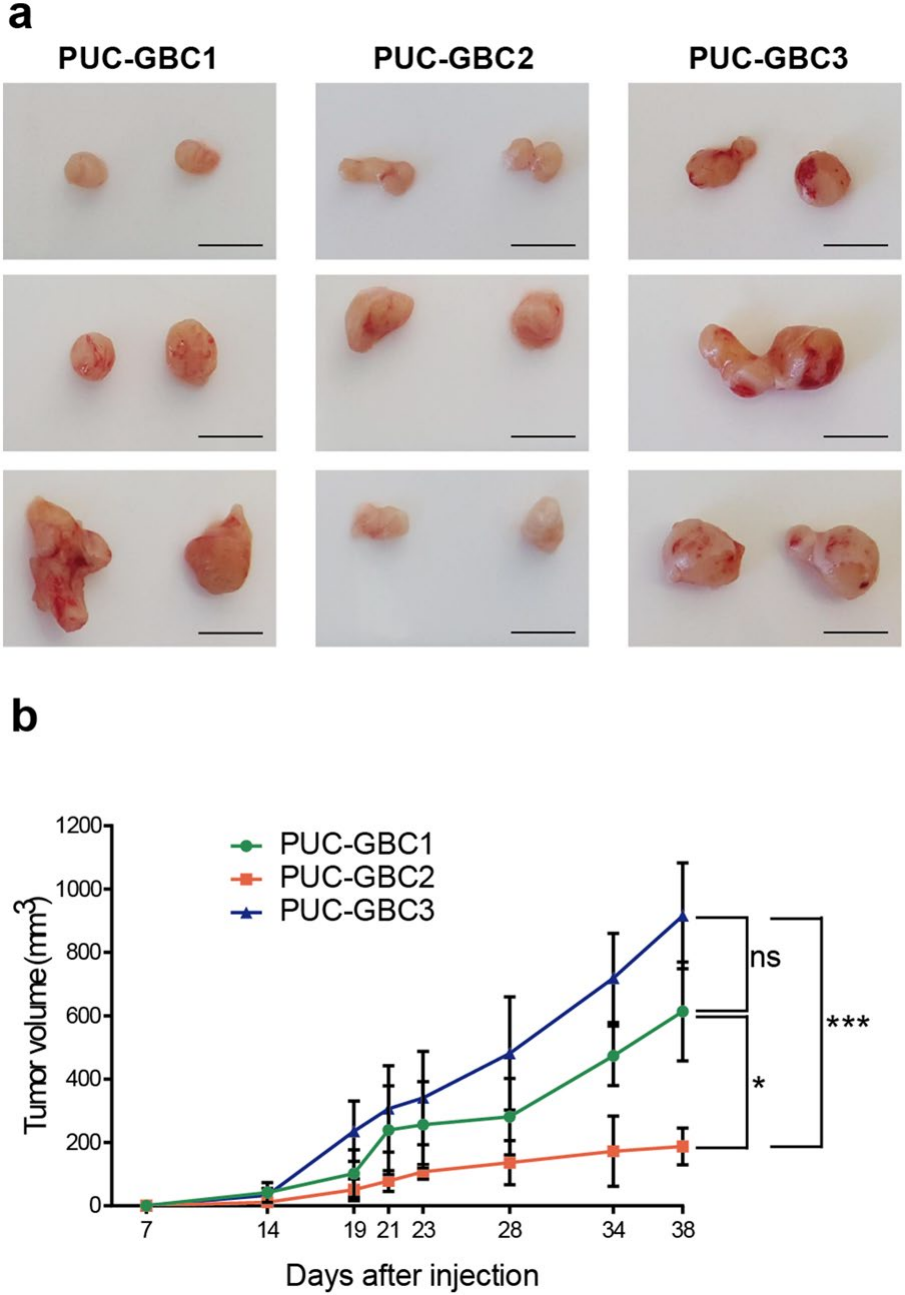
Tumorigenicity of ascites-derived gallbladder cancer cell lines. **a** Subcutaneous tumors induced by transplantation of the tumor cells (3 × 10^6^ cells) in NSG™ mice (scale bar, 1 cm). **b** Tumor growth curves of the subcutaneous mouse xenografts. Results are expressed as mean ± SD (n = 3 mice per group, *P = 0.031 and ***P = 0.0002 by Kruskal–Wallis with Dunn’s post-test at day 38)

In vivo tumorigenicity assays revealed palpable tumors in all mice within 15 days. As shown in Fig. 8, PUC-GBC3 exhibited the highest growth kinetics, followed by PUC-GBC1, whereas PUC-GBC2 had the lowest tumor-growing potential comparing to PUC-GBC3 (*P* = 0.0002) and PUC-GBC1 (*P* = 0.031) at day 38.

**Fig. 9:**
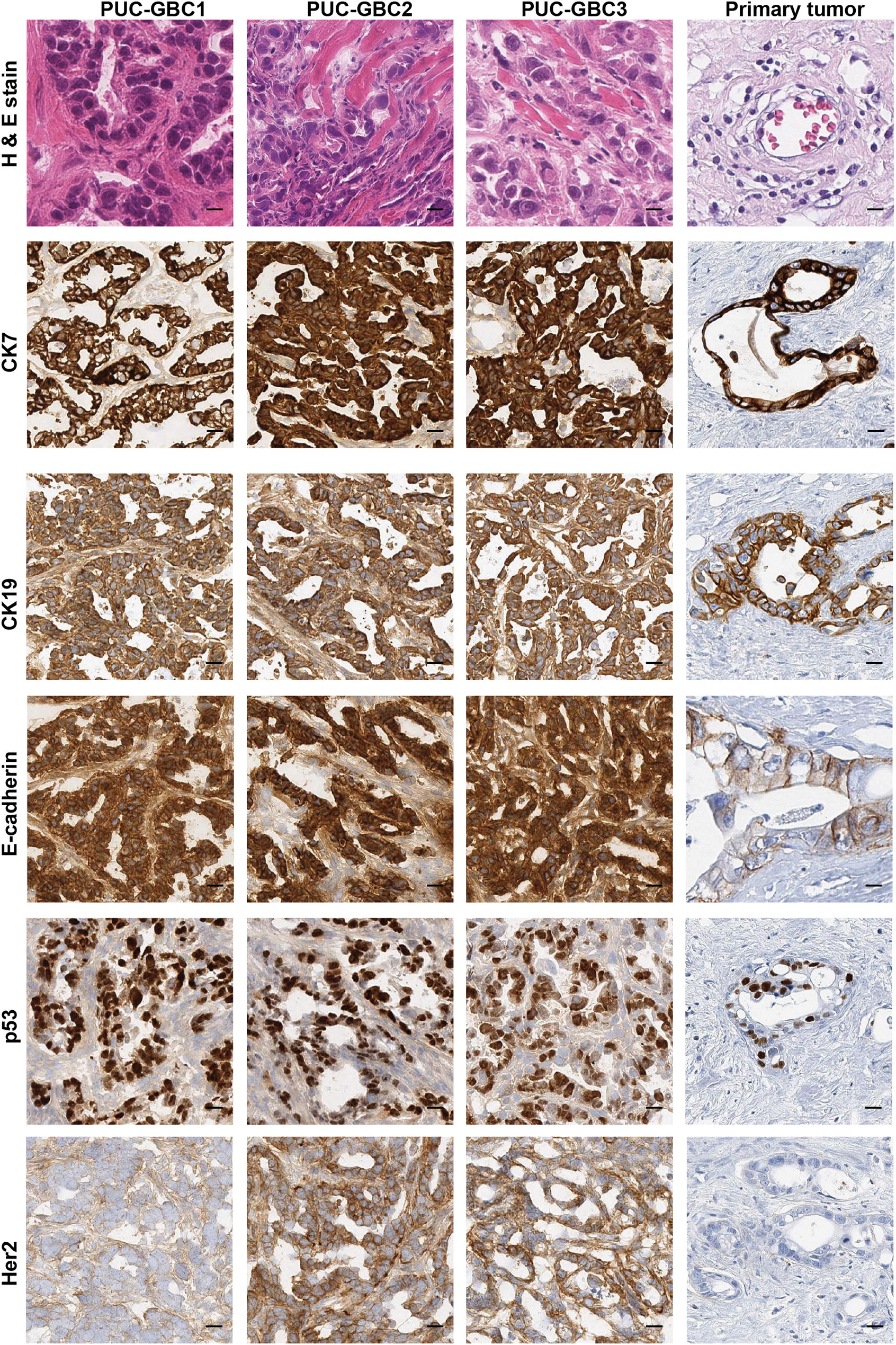
Xenograft tumors display high similarities with the original primary tumor. Xenografts showed histological features of adenocarcinoma, with tumor infiltration of muscle tissue. Expression of all markers was observed in the tumor cells of xenografts, except for HER2, which was present with different intensity only in cells derived from peritoneal metastasis. Scale bar, 20 µm; magnification, ×20

The hematoxylin- and eosin-stained tissue sections of the mice tumor xenografts showed histological features of adenocarcinoma, with high mitotic index, anisokaryosis and anisocytosis. Particularly, PUC-GBC1 and PUC-GBC2 xenografts were histologically similar and characterized by the presence of tubular structures constituted by cells with enlarged, hyperchromatic nuclei, frequent mitotic activity, abundant apoptotic bodies, and murine stroma dominating the tumor mass. In contrast, PUC-GBC3 tumors had a more aggressive appearance, with a more solid growth and less gland formation; also, isolated signet ring cells and scarce infiltration of the host stroma were noted (Fig. 9).

Immunohistochemistry (IHC) showed a strong positivity of the epithelial markers CK7, CK19 and E-cadherin (3+, 100% of the cells), which were also expressed in the primary tumor (Fig. 9 and Additional file 2 with RNA sequencing expression results). Nuclear positive p53 expression was observed in more than 70% of the cells in the xenografts in agreement with the expression observed in the primary tumor (Fig. 9). We also decided to evaluate the expression of human epidermal growth factor receptor 2 (HER2) because there is a subset of HER2-positive patients who might be candidates for an anti-HER2 therapy [27, 28]. We did not observe HER2 expression in the tissue section of the primary tumor or in the xenograft model of PUC-GBC1. However, PUC-GBC2 and PUC-GBC3 showed moderate and high HER2 intensity, and RNA sequencing revealed an increased *ERBB2* expression for all GBC cell lines, and particularly PUC-GBC1 and PUC-GBC2 (Additional file 2).

## Discussion

We report in this study on the phenotypic, genomic and functional characteristics of three new GBC cell lines derived from the metastatic ascites of a Chilean male patient; these were named PUC-GBC1, PUC-GBC2 and PUC-GBC3. Mapuche Native American ancestry has been strongly associated with the risk of GBC, and we found that the percentage of Mapuche Native American ancestry of the male donor of the primary tumor cells was 36%. For comparison, the first (third) quartiles of Mapuche ancestry reported in a recent study that included 1805 Chileans were 28% (43%) [6], confirming the representativeness of the established GBC cell lines for the Chilean population. The three new cell lines displayed an epithelial phenotype and showed abnormality in structure and number of chromosomes, with a tendency to triploidy. The doubling time varied from 36 to 60 h and was very similar to that observed for commercially available Asian cell lines. Evaluation of chemosensitivity indicated that our ascites-derived lines are more chemosensitive to gemcitabine and cisplatin than Asian GBC cell lines, which could be due to differences in molecular patterns. Furthermore, PUC-GBC1, PUC-GBC2 and PUC-GBC3 were tumorigenic when injected subcutaneously in NSG™ mice. Interestingly, the tumor volume of the PUC-GBC2 xenografts was the lowest despite PUC-GBC2 cells having the shortest doubling time and a higher migration capacity than PUC-GBC1 and PUC-GBC3 cells. In vivo, PUC-GBC3 cells showed a more aggressive behavior in agreement with the histopathological characteristics of the derived xenografts. The analysis of RNA sequencing data revealed differentially expressed genes that could be responsible for the phenotypic differences observed between the three cell lines. For instance, PUC-GBC2 showed elevated expression of *COL3A1*, which encodes an extracellular matrix (ECM) protein (collagen α‑1(III)) associated with tumor progression [29, 30] and has been reported as a marker of poor prognosis [31–33]. At the functional level, recent reports have demonstrated that COL3A1 promotes cell proliferation and migration [34, 35]. Similarly, high expression of *POSTN*, also overrepresented in PUC-GBC2, might explain the higher migratory ability of these cells. *POSTN* encodes for periostin, a multifunctional ECM protein that contributes to the remodeling of the tumor microenvironment during tumor progression [36]. In CCA and hepatoblastoma, overexpressed periostin was found to induce cell migration and epithelial-to-mesenchymal transition (EMT) features [37, 38]. On the other hand, PUC-GBC1 cells exhibited a slower growth rate and an enhanced in vivo tumorigenic capability. The transcriptome profile showed a high expression of genes related with cancer stem cell properties, such as *HNF1A*, a transcription factor recently reported to be enriched in pancreatic cancer stem cells [39] and a key player in the transcriptional reprogramming of colorectal cancer cells that promote liver metastasis [40]. RNA-Seq also confirmed the increased expression of cytokeratin and mucin genes in all three cell lines, showed an elevated *TP53* and *ERBB2* expression for PUC-GBC1 and PUC-GBC2, and revealed a novel exonic mutation in *NF1*.

We confirmed the epithelial origin of the primary culture and the derived cell lines by evaluation of the immunocytochemical expression of CK7 and CK19 (Additional file 1 and Fig. 9). Keratins are a complex subclass of the intermediate filaments, made up of more than 20 different polypeptides. Among them, CK19 belongs to the type I group of cytokeratins and is specifically expressed in the periderm, whereas CK7 is a type II cytokeratin specifically expressed in the simple epithelia lining the cavities of the internal organs and in the gland ducts and blood vessels [41]. Both CKs have shown normal expression in gallbladder epithelium [42] and are considered markers of biliary tract tumors [43].

We also evaluated the expression of CA 19-9, which is a glycolipid synthesized by the pancreatic, biliary, gastric, colonic cells, as well as the endometrial and salivary epithelia [43]. We found elevated CA 19-9 levels in almost all the cells of the primary culture and of the clones PUC-GBC1 and PUC-GBC3. The intensity of CA 19-9 expression was also high in PUC-GBC2, but only in 20% of cells (Fig. 2). Recently, Barnett et al. reported that differential expression of CA 19-9 and sTRA (Sialyl-TRA), another carbohydrate antigen, made it possible to identify morphologically distinct subsets of pancreatic cancer cells. Subpopulations expressing both markers were part of well differentiated and mucin-secreting pancreatic adenocarcinomas, whereas those expressing just one were often poorly differentiated and vacuolated and never mucin-secreting. In addition, the authors observed cases displaying sometimes more than one subpopulation in the same tumor [44]. A heterogeneous distribution of CA 19-9 expression in GBC could explain our findings, especially considering that the clones were isolated from malignant ascites, characterized by containing a heterogeneous group of tumor cells. However, more research is needed to determine if cells with different patterns of CA 19-9 expression co-exist in gallbladder tumors.

Other tumor markers strongly expressed by the primary culture and individual clones were the mucins MUC1 and MUC16, particularly MUC1 according to RNA sequencing results (Additional file 2). MUC1 and MUC16 are transmembrane glycoproteins, located in 1q22 and 19p13 locus respectively, which contribute to forming physiological barriers and transmitting growth and survival signals to the cells [45]. High MUC1 immunoreactivity has been associated with vascular invasion and the malignant progression of many tumors [46–51], including GBC [52, 53]. Studies have reported that MUC1 expression is higher in GBC than in normal and inflammatory gallbladder tissue [54–57] and more frequently and extensively expressed in poorly differentiated adenocarcinoma [58]. The upregulation of MUC1 in cancer is a product of the aberrant O-linked glycosylation that lead to an increased sialylation of this protein [59, 60]. Interestingly, the expression rate of stromal localization of sialylated MUC1 at the deepest invading sites of pT2 gallbladder carcinoma seems to be associated with a more frequent postsurgical peritoneal dissemination and lower survival rate of these patients [54]. Similar findings have reported that sialylated MUC1 mucin plays an important role in the progression of prostate cancer [61] and may be involved in the metastatic potential of pancreatic ductal adenocarcinoma [62].

On the other hand, the upregulation of MUC16 in primary tumor and ascites-derived clones could be associated with a biologically aggressive phenotype (Figs. 1, 2). Indeed, MUC16 (cancer antigen 125, CA 125) is used in clinics to diagnose and predict prognosis in ovarian cancer patients, but it is also upregulated in a large percentage of digestive tract adenocarcinomas and has been proposed as a prognostic marker for gastrointestinal cancers [63]. In a recent study, positive immunohistochemical staining of MUC16 (immunoreactivity in more than 10% of the tumor cells) was observed in more than 75% of extrahepatic pancreatobiliary tumors (n = 234 cases), including 37 gallbladder cancers [64]. Chaube et al. proposed MUC16 as a potential diagnostic marker because serum levels are increased in patients with GBC and benign disease can be differentiated from malignant disease with a sensitivity of 64% and a specificity of 90% [65].

These newly established cell lines, in particular PUC-GBC1 and PUC-GBC2, showed overexpression of p53, which was also observed in the primary tumor and xenografts (Fig. 9). In GBC, most studies have reported a frequency over 50% of p53 overexpression [20, 23, 66–68]. In addition, there is a progressive increase in p53 positivity from dysplastic lesions to carcinoma in situ and invasive carcinoma [23, 66, 69], and a high correlation between protein overexpression and the presence of *TP53* mutations [20, 21]. The cell lines described here conserved the p53 overexpression observed in the primary tumor, although our analysis did not show any *TP53* mutation. It has been generally accepted that mutations in *TP53* may result in the synthesis of a functionally defective p53 protein that exhibits an extended half-life, which tends to accumulate in the cell nucleus and can be detected by immunohistochemistry [70, 71]. However, studies in other tumors have described immunohistochemical overexpression of p53 in absence of gene mutations due to the deregulation of upstream factors of p53 pathway, which lead to protein stabilization and accumulation in the nucleus [72, 73]. Based on our data, it is highly probable that the p53 signaling pathway, along with other relevant oncogenic pathways, is dysfunctional in all three cell lines. For instance, genomic analysis revealed a nsSNV in *TP53* (dbSNP identifier: rs121912651) in all cell lines, which has not been reported as an oncogenic mutation but likely possesses a pathogenic role according to the ClinVar database (https://www.ncbi.nlm.nih.gov/clinvar/). Additionally, we found a nsSNV in *MDM4* and a frameshift deletion in *CDKN2A*, both genes encoding proteins related with regulation of p53 (Additional file 3). However, further studies are needed in order to determine the level of p53 activity in these novel cell lines, as well as the functional and clinical significance of those genetic variants in gallbladder cancer.

We also assessed the expression of HER2 in primary tumors and xenografts. Overexpression of this protein has been reported in 14–48% of GBC cases [27, 28, 74–78], and its amplification and mutation has been observed in 5–20% and 10% of the cases, respectively [27, 75–77, 79]. The newly established GBC cell lines did not present any *ERBB2* mutation. HER2 upregulation has been associated with poor prognosis in GBC [77, 78], and experimental tumor models have suggested that HER2 signaling is involved in gallbladder carcinogenesis [80, 81]. In this study, immunohistochemistry for HER2 was negative for the primary tumor and PUC-GBC1 xenografts but was 2+ for PUC-GBC2 and 3+ for PUC-GBC3 (Fig. 9). *ERBB2* was particularly highly expressed in PUC-GBC1 and PUC-GBC2, and showed a somewhat lower expression in PUC-GBC3. This is not an unexpected finding considering that intratumor heterogeneity is a common event in cancer, and genetic heterogeneity of HER2 has been reported for other tumors [82–88]. For instance, focal or patchy positivity of HER2 is a pattern encountered in primary gastric tumors, which is consequence of the intratumor heterogeneity and could explain discordances observed in HER2 status between the primary tumor and metastatic sites [82, 85]. Recently, Toshiba et al. investigated the status of HER2 in a large cohort of patients with GBC and observed intratumor heterogeneity in 51% (20/39) of the cases with IHC scores of 2+ and 3+. Among them, around 80% showed amplification of HER2/neu gene as determined by fluorescent in situ hybridization (FISH) [27]. Based on these observations, it would be relevant to perform the immunohistochemical test for HER2 in primary and metastatic sites if clinical trials with anti-HER2 therapies are considered for patients with this disease. In this regard, it would be even more advisable to implement the technology of liquid biopsies and take advantage of their clinical value for the identification of heterogeneous subclonal populations of tumor cells.

## Conclusions

In summary, we established and characterized three new GBC cell lines derived from a patient with metastatic disease, who represents well the Chilean population with a 36% percentage of Mapuche Native American ancestry. The new cell lines share some biological characteristics, but also show genomic and phenotypic differences that reflect intratumor heterogeneity. The established GBC cell lines provide a new experimental model for future research, including the study of cellular and molecular mechanisms involved in metastasis, the identification of new tumor-associated markers, and the evaluation of responses to new therapeutic agents.

## Materials and methods

### Cell lines and growth conditions

A panel of five cell lines of Asian origin was used for comparison of doubling times and drug sensitivity. GB-d1 [14] was provided by Dr. Anirban Maitra (Department of Pathology, Division of Pathology and Laboratory Medicine, The University of Texas MD Anderson Cancer Center, Houston, TX, USA); NOZ was obtained from the Health Science Research Resources Bank (Osaka, Japan; No JCRB1033); and G-415, TGBC-1TKB and TGBC-2TKB were purchased from RIKEN BioResource Center (Ibaraki, Japan; No RCB2640, RCB1129 and RCB1130). G-415 and GB-d1 were grown in RPMI 1640 medium (Thermo Scientific HyClone, Logan, UT, USA) supplemented with 10% fetal bovine serum (FBS), 10 units/mL penicillin and 10 mg/mL streptomycin (1% penicillin/streptomycin, Thermo Scientific HyClone). NOZ, TGBC-1TKB and TGBC-2TKB were grown in Dulbecco’s Modified Eagle Medium (DMEM high glucose; Corning, New York, NY, USA) supplemented with 5% FBS and 1% penicillin/streptomycin.

### Isolation and establishment of human gallbladder cancer cell lines

The primary cell culture was established from the ascites of a 60-year-old male patient with GBC. Histological examination classified this tumor as moderately differentiated tubular adenocarcinoma with a T3NxM1 stage. There was evidence of vascular and lymphatic system invasion and peritoneal carcinomatosis. Ascitic fluid (50 mL) was obtained from the patient, delivered to the laboratory and centrifuged at 1000 rpm for 10 min, and the pellet was rinsed twice with sterile phosphate-buffered saline (PBS; Corning, New York, NY, USA) containing antibiotics (1% penicillin–streptomycin solution, P/S). The supernatant was removed and saved as ascitic fluid supplement at -20 °C, and the remaining cells were resuspended in DMEM/F12 medium (HyClone, GE Healthcare Bio-Sciences, Pittsburgh, PA, USA) supplemented with 5% FBS, 50% of ascitic fluid (HyClone, GE Healthcare Bio-Sciences, Pittsburgh, PA, USA) and 1% P/S, seeded into 6-well culture plates, and incubated at 37 °C in a humidified atmosphere of 5% CO_2_ in the air. The growth medium was replaced every 2–3 days, and the plate was regularly checked for epithelial cells and fibroblast outgrowth. The ascitic fluid supplement was maintained in the culture and reduced 10 times every week until the cells reached confluency. After the first subculture, the cells were grown in complete culture medium alone (without added ascitic fluid) and submitted to immunocytochemistry analysis to confirm the epithelial origin and evaluate the expression of tumor markers. This analysis was repeated at passage 17. Primary culture cells were subcultured until they reached more than 20 passages. Then, three individual clones were obtained by serial dilution of the primary cell culture in a 96-well plate. Once established, all cell lines were cultured in DMEM supplemented with 5% FBS and 1% P/S. All cultures were tested for mycoplasma contamination with a commercial PCR kit (EZ-PCR Mycoplasma Test Kit, Biological Industries, Cromwell, CT, USA).

### Immunostaining procedures

Primary antibodies and working dilutions used for immunostaining analysis were mouse monoclonal anti-cytokeratin 7 (CK7; Cat. No M7018; Clone OV-TL 12/30) and mouse monoclonal anti-p53 (Cat. No IS616: Clone DO-7) from Dako (Agilent Technologies, Santa Clara, CA, USA); mouse monoclonal anti-mesothelin (Cat. No ACI 3175; Clone MSLN-15C11) from Biocare Medical, LLC (Pacheco, CA, USA); mouse monoclonal anti-CA 15-3 (MUC1; Cat. No MU323-UC; Clone BGX323A) from Biogenex (Fremont, NE, USA); mouse monoclonal anti-cytokeratin 19 (CK19; Cat. No 760-4281; Clone A53-B/A2.26), mouse monoclonal anti-CA 19-9 (Cat. No 760-2609; Clone 121SLE), mouse monoclonal anti-CA 125 (MUC16; Cat. No 760-2610; Clone OC125), rabbit monoclonal anti-calretinin (Cat. No 790-4467; Clone SP65), mouse monoclonal anti-vimentin (Cat. No 790-2917; Clone V9), mouse monoclonal anti-E-cadherin (Cat. No 790-4497; Clone 36) and rabbit monoclonal anti-HER2 (Cat. No 790-2991; Clone 4B5) from Roche Tissue Diagnostics (Oro Valley, AZ, USA).

The epithelial and tumor origin of the primary culture from the ascites and the derived cell lines was evaluated by immunocytochemistry. Cells were seeded at 2 × 10^5^ per well in 24-well plates and allowed to attach for 24 h. Following fixation in 4% paraformaldehyde for 15 min, PBS wash and permeabilization with 0.1% Triton X-100, cells were incubated with primary antibodies at 1:100 dilution for 1 h. Then, cells were washed four times with 0.2% BSA (bovine serum albumin) in PBS and incubated for 30 min with the SuperPicture™ Polymer Detection Kit (Thermo Fisher Scientific Inc., Waltham, MA, USA). The bound antibody was visualized with a red chromogen (ImmPACT™ NovaRED™ Substrate; Vector Laboratories Inc., Burlingame, CA, USA). Antibody for HER2/neu was immunostained on the Benchmark XT automated stainer (Ventana Medical Systems, Tucson, AZ, USA) according to the manufacturer’s instructions. Images were acquired using the EVOS XL Core Imaging System (Thermo Fisher Scientific Inc., Waltham, MA, USA).

The primary tumor and xenografts were analyzed immunohistochemically on formalin-fixed paraffin-embedded (FFPE) sections (2 μm thickness) using the automated immunostainer BenchMark ULTRA and the ultraVIEW universal DAB detection kit (Ventana Medical Systems Inc., Roche Group, Tucson, AZ, USA). Digital images were captured using the Aperio AT2 Digital Pathology Scanner (Leica Biosystems, Nussloch, Germany). The expression was evaluated by the pathologist (J.C.R.) considering the intensity of the staining (1 + , weak; 2 + , moderate; 3 + , strong) and the percentage of labeled cells. Immunohistochemical scoring of HER-2 expression was based on the system proposed for gastric and gastroesophageal junction cancer [89].

### Short tandem repeat (STR) DNA profiling

The genetic profiling of the cell lines derived from the primary cell culture was established using the polymorphic STR loci detection service offered by the Department of Molecular Medicine at Aarhus University Hospital (IdentiCell service for human cell line authentication). DNA of each cell line was purified using the PureLink^®^ Genomic DNA mini kit (Thermo Fisher Scientific Inc., Waltham, MA, USA).

### Chromosome analysis

The cytogenetic karyotyping analysis was performed using a standard air-dried method. Briefly, cells in an exponential growth phase were treated with a final concentration of 0.1 µg/mL colcemid (Colcemid^®^ Solution, Irvine Scientific, Santa Ana, CA, USA) for 45 min at 37 °C. Then, cells were harvested to arrest metaphases and treated with the hypotonic solution (0.075 M KCl) for 10 min at 37 °C. After two changes in the fixative (3:1, methanol: glacial acetic acid), the cell suspension was incubated overnight at -20 °C. The next day, the fixative was changed another three times and two drops of the cell suspension were dropped from a distance of about 50 cm onto clean dry slides tilted at an angle of about 45°, allowing the cells to roll across the slide. After air drying, one slide was stained with Giemsa (3:1 ratio of Gurr Buffer and Giemsa Stain) and analyzed under a light microscope at 10× and 100× magnification. If the metaphase cells were abundant and well spread, the remaining slides were used for chromosome analysis using trypsin G-banding. To determine ploidy, chromosomes were counted from a minimum of 50 metaphase spreads using GenASIs Karyotyping (Applied Spectral Imaging Inc. Carlsbad, CA, USA).

### Cell growth characteristics

The doubling time was calculated by seeding the cells at a density of 20 000 cells/well in 12-well plates and counting them by Trypan blue dye exclusion in a Neubauer camera every 24 h for 15 days (the medium was replaced every 3 days). The doubling times were determined from the growth curves using the data generated from 3 independent experiments, each with three technical replicates.

### Chemosensitivity assay

The cytotoxic drugs used for in vitro chemosensitivity assays were gemcitabine (Sigma-Aldrich, St. Louis, MO, USA), cisplatin (Calbiochem, Merck group, Darmstadt, Germany) and Fluorouracil (Laboratorios Kampar, Santiago, Chile). These drugs are the main chemotherapy agents used to treat GBC. Cells were seeded into 96-well plates at a density of 5 × 10^3^ cells/well in 100 μL cell culture medium. After an overnight attachment period, cells were treated with either gemcitabine (starting at 300 µM), cisplatin (starting at 350 µM) or fluorouracil (starting at 2 mM) with a serial dilution of 1/3. Following 72 h of drug incubation, cell viability was assessed by incubating the cells for 2 h at 37 °C with a MTS-PMS colorimetric solution (Promega Corp., Madison, WI, USA), and absorbance of each well was read at a wavelength of 490 nm using a multiwell plate reader (Epoch Microplate Spectrophotometer, BioTek Instruments Inc., Winooski, VT, USA). The half maximal inhibitory concentration was calculated from the dose response curves (IC_50_: dose of drug required to reduce final cell number or optical density in MTS assay to 50% of control). Three independent experiments were performed, each with three technical replicates.

### Cell migration assay

Migration assays were performed using Transwell^®^ 24-well plates containing polycarbonate filters with an 8-micron pore size (BD Biosciences, Bedford, MA, USA). Complete medium was placed in the lower chamber to act as a chemoattractant, and 5 × 10^4^ cells were seeded with serum-free medium into the upper chamber. After 24 h, cells were fixed in methanol for 15 min and stained with 0.05% crystal violet in 25% methanol/PBS for 15 min. Cells on top of the membrane were removed using a cotton swab, and filters were washed with PBS. Cells on the underside of filters were viewed and counted under a microscope in 10 randomly selected fields. Three independent experiments were performed, each with three technical replicates.

### Tumorigenicity assay

The in vivo tumorigenicity assay was performed using 7 to 8-week-old male NOD *scid* gamma (NSG™) mice obtained from The Jackson Laboratory (Bar Harbor, ME, USA). The animals (n = 9) were randomized into three groups, anesthetized with isoflurane (3% for induction of anesthesia and 1.5% for maintenance) and 3 x 10^6^ cells suspended in 150 μL PBS/matrigel (Matrigel™ Basement Membrane Matrix, BD Biosciences, Bedford, MA, USA) were injected bilaterally and subcutaneously on the back of the anesthetized mice. Animals were examined every week for the development of tumors. Tumor volumes were estimated twice a week from caliper measurements (volume = 0.52 × (width)^2^ × length). When tumors had grown to 1.5–2.0 cm^3^ after 4 weeks, mice were euthanized by carbon dioxide (CO_2_) and the tumor xenografts were removed, weighted and photographed. Then, tumors were fixed in 10% neutral buffered formalin, and processed for routine histopathological examination.

### Variant and gene expression analysis from RNA sequencing, and estimation of genetic ancestry proportions

Total RNA was extracted and sequenced on an Illumina Hi-Seq 2000. The quality of the sequencing reads was assessed using FastQC (0.11.2) (http://www.bioinformatics.bbsrc.ac.uk/projects/fastqc). PRINSEQ (0.20.3) [90] and cutadapt (1.9) [91] were used to filter the reads based on quality parameters and to remove adapters. Variant calling was performed following the GATK guidelines for RNA sequencing data, including a two-pass alignment with STAR (2.5.2b) [92], towards the GRCh38 version of the human genome, removal of PCR duplicates with picard tools (2.1.0) (http://broadinstitute.github.io/picard/) and variant calling with GATK 3.5 [93]. Thresholds for the detection of mutations were read depth > 20, allelic depth > 10, mapping quality > 40 and Fisher’s strand < 60. Variants were annotated with Annovar [94]. Gene expression values were calculated using feature Counts from the subread package (1.6.4) [95] and normalized as transcripts per million. More than 30 M reads were generated per each cell line sample. Additional file 5 provides the sequencing statistics for the three newly established GBC cell lines. Gene ontology analyses were conducted with the R package goProfiles.

The identified variants with an allele frequency > 5% according to the 1000 Genome Project (more than 5000 variants in total) were used to infer the genetic ancestry proportions of the donor of the primary GBC tumor cells [96]. Surrogates of African and European ancestry were 87 Yorubans in Ibadan, Nigeria, and 80 Utah residents with Northern and Western European ancestry from the 1000 Genome Project. Nine Mapuche and nine Aymara individuals were selected to represent the two largest indigenous peoples in Chile based on the three following criteria: four grandparental Mapuche or Aymara surnames, estimated Native American proportion of at least 74% for Mapuche and at least 99% for Aymara reference individuals, and mitochondrial DNA haplogroups consistent with Mapuche (haplogroup C or D) or Aymara (haplogroup B) descent [6]. The ADMIXTURE software was used for supervised estimation of the African, European, Mapuche Native American and Aymara Native American ancestry components relying on the above-mentioned identified common variants, reference individuals and genotype data from a recent study that included 1805 Chileans [6, 97].

### Statistical analysis

Statistical analyses were performed using R programming environment in Rstudio© version 1.0 (Rstudio, Inc., Boston, MA). A Kruskal–Wallis test was used to compare multiple groups, followed by Dunn’s post hoc multiple comparisons test. Probability values smaller than 0.05 were considered statistically significant.

## Supplementary information

**Additional file 1.** Representative micrographs of immunocytochemical staining for CK7, CK19, Mesothelin, Vimentin and Calretinin in the three clones isolated from the ascites-derived primary culture (magnification, 40×).

**Additional file 2.** Gene expression of normalized transcripts per million in Chilean GBC cell lines.

**Additional file 3.** Exonic mutations in Chilean GBC cell lines.

**Additional file 4.** Venn diagrams depicting unique and shared (overlapping circles) variants in the three cell lines. (a) non-synonymous; (b) synonymous; and (c) frameshift. The green circle depicts the variants identified in PUC-GBC1, the orange circle depicts the variants identified in PUC-GBC2, and the blue circle depicts the variants identified in PUC-GBC3.

**Additional file 5.** Sequencing statistics.

## Data Availability

All data generated and/or analyzed in this study are included in this published article and its additional files. Additionally, they are available from the corresponding author on reasonable request.

## Abbreviations

GBC: Gallbladder cancer
CK7: Cytokeratin 7
CK19: Cytokeratin 19
CA 19-9: Carbohydrate antigen 19-9
MUC1: Mucin 1
MUC16: Mucin 16
TP53: Tumor Protein P53
STR: Short tandem repeat
GO: Gene ontology
nsSNV: Nonsynonymous single-nucleotide variation
SIFT: Sorting intolerance from tolerance
5-FU: Fluorouracil
HER2: Human epidermal growth factor receptor 2
ERBB2: Erb-B2 Receptor Tyrosine Kinase 2
IHC: Immunohistochemistry
CKs: Cytokeratins
sTRA: Sialyl-TRA
CA 125: Cancer antigen 125
FISH: Fluorescent in situ hybridization
DMEM: Dulbecco’s Modified Eagle Medium
FBS: Fetal bovine serum
PBS: Phosphate-buffered saline
P/S: Penicillin-streptomycin solution
BSA: Bovine serum albumin
FFPE: Formalin-fixed paraffin-embedded
NSG: NOD scid gamma
