# Supplementary figures and images for: Functional and genomic characterization of three novel cell lines derived from a metastatic gallbladder cancer tumor
Source: Biol Res. 2020 Apr 15;53:13. doi: 10.1186/s40659-020-00282-7 (PMC7158131; doi:10.1186/s40659-020-00282-7)

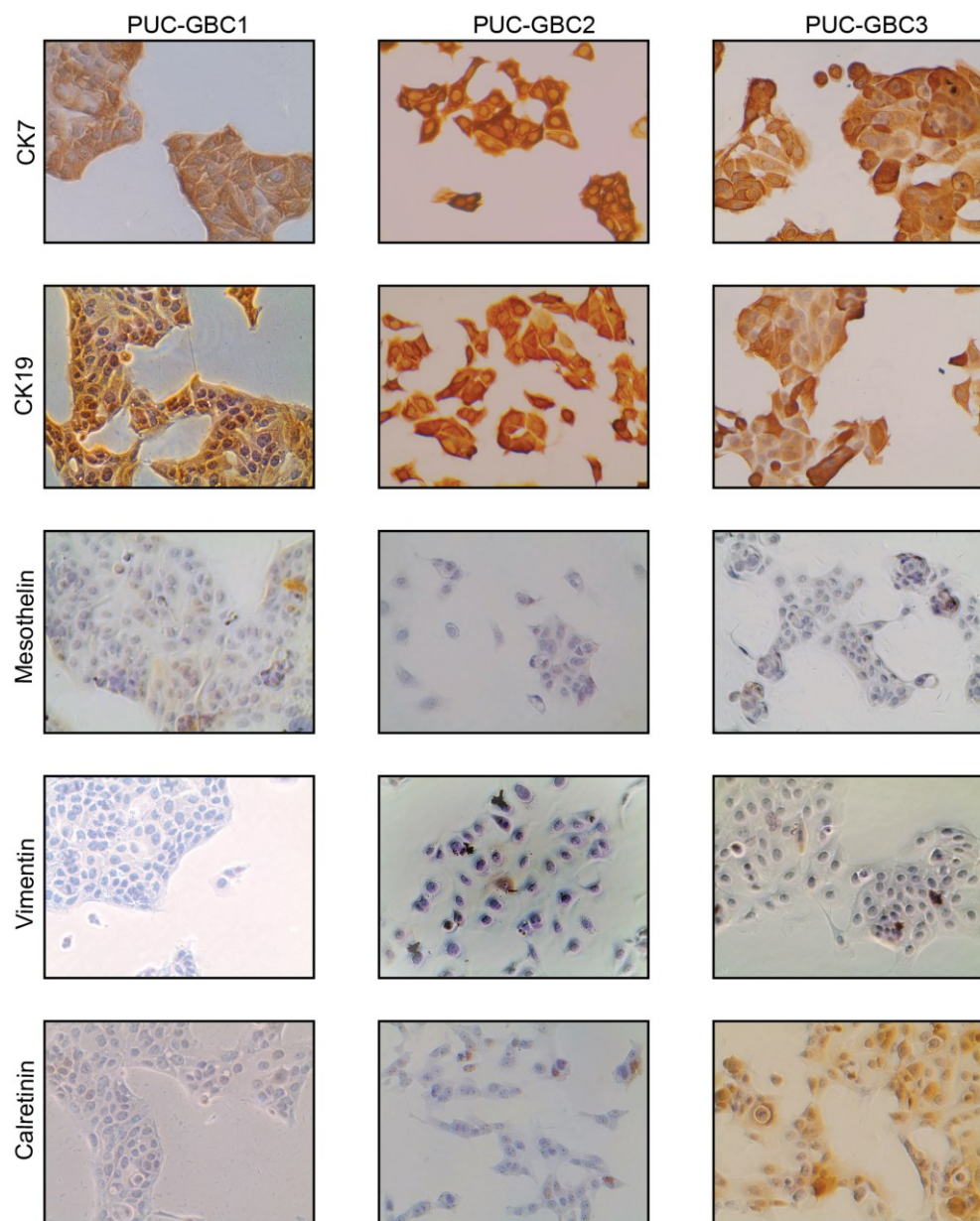

Supplement: Supplementary file 1 — Additional file 1. Representative micrographs of immunocytochemical staining for CK7, CK19, Mesothelin, Vimentin and Calretinin in the three clones isolated from the ascites-derived primary culture (magnification, 40×). [file 40659_2020_282_MOESM1_ESM.pdf]

**a**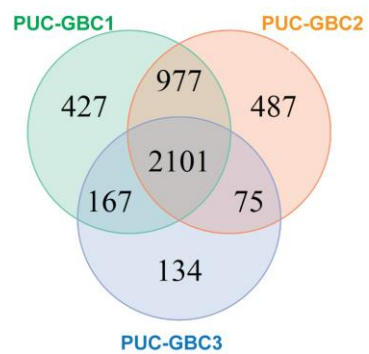**b**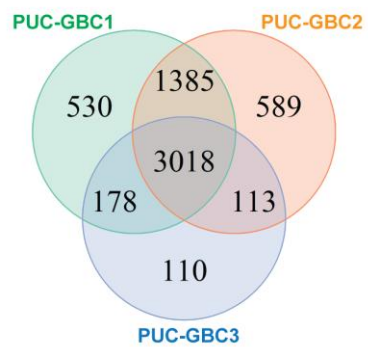**c**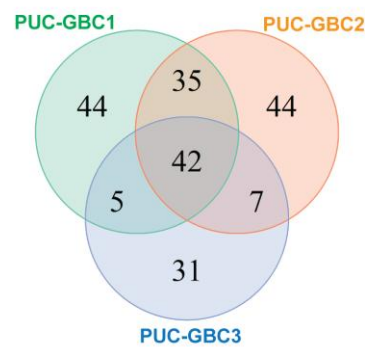

Supplement: Supplementary file 4 — Additional file 4. Venn diagrams depicting unique and shared (overlapping circles) variants in the three cell lines. (a) non-synonymous; (b) synonymous; and (c) frameshift. The green circle depicts the variants identified in PUC-GBC1, the orange circle depicts the variants identified in PUC-GBC2, and the blue circle depicts the variants identified in PUC-GBC3. [file 40659_2020_282_MOESM4_ESM.pdf]
